# Meta-analysis of the intervention effects of taekwondo on metabolic syndrome indicators

**DOI:** 10.3389/fphys.2023.1069424

**Published:** 2023-01-17

**Authors:** Zhengfa Han, Hanyu Ju

**Affiliations:** ^1^ Department of Physical Education, Yongin University, Yongin, South Korea; ^2^ Department of Sports Science, Kyonggi University, Suwon, South Korea

**Keywords:** metabolic syndrome, intervention effect, meta-analysis, intervention method, taekwondo

## Abstract

**Objective:** To quantify the effect of taekwondo as an intervention on the indicators of metabolic syndrome and identify an intervention plan with the optimal effects.

**Methods:** Combining the Cnki.net, Wanfang, PubMed, Web of Science, Embase, KISS, RISS, and DBPIA databases, this paper retrieved relevant references in Chinese, English, and Korean, applied Review Manager 5.4 software to evaluate the methodological quality of the included references according to the Cochrane manual, and utilized Comprehensive Meta-Analysis version 3.7 to perform statistical analyses.

**Result:** A total of 45 references and 1079 related subjects were included in the analysis. The results of the meta-analysis showed that taekwondo has a beneficial effect on all indicators of metabolic syndrome (Hedges’ g [effect size] = −0.615, −0.672, −0.497, −0.785, −0.591, and 0.435; *p* < 0.05). Subgroup analysis revealed the superior intervention effect of taekwondo on metabolic syndrome in women compared to men, middle-aged and elderly compared to other age groups, and abnormal metabolic syndrome indicators compared to normal values. Moreover, the best results were obtained for longer intervention durations—12 weeks—three times per week, for 40–50 min per session. In addition, the combination of intervention types (poomsae, kick, and taekwondo gymnastics) showed optimal effects. The exercise intensity should consider the characteristics of the intervention object and be generally set to medium or high intensity.

**Conclusion:** Taekwondo can effectively improve metabolic syndrome, as evidenced by decreased body mass index (BMI), systolic blood pressure (SBP), diastolic blood pressure (DBP), fasting blood glucose (FBG), and triglyceride (TG) levels and increased high-density lipoprotein cholesterol (HDL-C) level. Taekwondo had the greatest effect on quinquagenarian women with abnormal levels of metabolic syndrome indicators. To maximize the intervention effect of taekwondo on metabolic syndrome, an exercise prescription of high-intensity poomsae, kick, and taekwondo gymnastics performed in 40–50 min sessions, three times weekly for 12 weeks is recommended.

**Clinical Trial Registration:** https://www.crd.york.ac.uk/prospero/, identifier CRD42022362495

## 1 Introduction

Metabolic syndrome (MS) is the physiological and metabolic accumulation of cardiovascular risk factors, as indicated by abdominal obesity and high triglyceride (TG), blood pressure (BP), fasting blood glucose (FBG), and high-density lipoprotein cholesterol (HDL-C) levels (Van Namen et al., 2019). Currently, the most frequently accepted criteria for MS are abnormal measures of three or more of the following: abdominal obesity (extremely important, but not required), triglyceride, blood sugar, blood pressure, and high-density lipoprotein cholesterol (Grundy, 2016). Due to poor nutrition and a lack of physical activity, MS has become a global epidemic in recent decades (Lim et al., 2008). MS has attracted attention due to its worldwide incidence and high correlation with diabetes and cardiovascular disease (Gluvic et al., 2017; Bauset et al., 2022). MS appears to have a minimal risk factor; however, it may not only cause numerous complications but also increase the morbidity and mortality of numerous chronic diseases (Ford, 2005). MS can increase the risks of cardiovascular disease and type 2 diabetes by twofold and fivefold, respectively (Grundy, 2016). MS has become a global epidemic requiring significant attention. O’Neill and O’Driscoll (2015) reported that the etiology of MS is highly related to physical activity and carbohydrate consumption. Multiple studies have also demonstrated that physical activity can reduce the risk of MS (Kelishadi et al., 2007).

Taekwondo has positive effects on obesity, BP, TG, FBG, HDL-C, and other MS indicators in addition to enhancing the physical fitness of participants (Kim and Kim, 2008; Park et al., 2010; Kim et al., 2021; Lee and Kim, 2021). However, some studies have reported that taekwondo has the opposite effect on MS indicators such as BMI, BP, TG, FBG, and HDL-C (Lee et al., 2017; 2018). Although researchers have conducted high-quality studies, whether taekwondo interventions have a positive effect on MS indicators remains controversial, and no meta-analysis has been conducted yet to address this topic. Consequently, questions such as “if Taekwondo intervention has a positive effect on MS, how substantial is the effect?” and “are the results of the intervention affected by the characteristics of the study object or the study methodology?” can be posed.

The present meta-analysis was performed to quantify the effect of taekwondo interventions on MS indicators and identify intervention plans that can produce the optimized intervention effect. These results will serve as a reference for future research on MS interventions.

## 2 Methods

This meta-analysis is based on the PRISMA 2020 checklist and was registered in PROSPERO (registration number: CRD42022362495).

### 2.1 Literature search

Chinese, Korean, and English databases, including CNKI, Wanfang Data, KISS, RISS, DBPIA, PubMed, Web of Science, and Embase databases, were searched to maximize literature retrieval. Articles that had been indexed as of 25 September 2022 were included. Using the “keyword” + “keyword” search strategy, we looked for terms using both their full names and their abbreviations. The detailed search strategies are included in the Supplementary Material. A total of 569 studies were retrieved, including 68 studies from the Chinese database, 173 articles from the Korean database, 328 articles from the English database, and 173 articles from the Chinese database.

### 2.2 Inclusion and exclusion criteria

Two researchers (ZH and HJ) separately searched the literature using the PICOS method. In cases of disagreement, a decision was reached after debate; in cases where discussion failed to produce a consensus, the supervising professor made the final call. The inclusion criteria were as follows: 1) unlimited research objects; 2) taekwondo training interventions of any kind, including poomsae, kick, and taekwondo gymnastics, other dietary interventions, and combined treatments were not included in the intervention group; 3) control groups performing regular activities according to everyday routines; 4) study indicators including BMI, SBP, DBP, FBG, TG, and HDL-C; and 5) randomized controlled studies (RCTs) study. Studies for which the effect size could not be calculated from the reported data were also eliminated. The data reported in the article only needed to meet one of the following conditions to calculate the effect size: 1) reported means, standard deviation (SD), and sample size (n) of the control and intervention groups; 2) reported differences in means, common SD, and sample size (n) between the control and intervention groups; 3) reported means and sample size (*n* and *T*) values of the control and intervention groups; 4) reported differences in means and sample size (n and T) values between the control and intervention groups; and 5) reported means, sample size (*n*), and *p*-values of control and intervention groups.

### 2.3 Data extraction

Two researchers independently completed data extraction (ZH and HJ). Duplicate references were first removed, and irrelevant studies were removed after reading the article’s title and abstract. The studies included in the meta-analysis were then strictly screened by reading the whole text for compliance with the inclusion criteria. Information such as the article title, first author, publication year, sample size, major result details, subject characteristics, intervention type, and other details was retrieved during the screening process. Disagreements during the data extraction process were resolved through conversation. The supervising professor made the final judgment if a consensus could not be achieved following the discussion.

### 2.4 Assessment of the risk of bias and publication bias

Two researchers (ZH and HJ) independently evaluated the selection bias conduct bias, measurement bias, follow-up bias, reporting bias, and other biases of each included study, using Review Manager 5.4 software according to the Cochrane Review manual. Each bias’s risk was divided into three categories: high, low, and unclear. Additionally, publication bias was assessed using the funnel plot and Egger’s test.

### 2.5 Statistical analysis

Review Manager 5.4 was used to assess the risk of bias. Comprehensive Meta-Analysis 3.7 was used to examine the remaining studies.

In statistics, the effect size is the value that quantifies the magnitude of a phenomenon, including but not limited to the association strength between two variables (Kelley and Preacher, 2012). The overall scores of the control and intervention groups were used to determine each effect size, and one or more effect sizes might be produced for each experimental study. The combined effect size in a meta-analysis is typically calculated using the mean difference value. The standardized mean difference (SMD) was selected as the merged effect size because the measuring tools used in the included studies differed. The most common SMD expression forms include Cohen’s d, Hedges’ g, and Glass’ s△. Cohen’s d will overestimate the effect size when the sample size is small (Borenstein et al., 2009). Hedges’ g was produced by standardized-mean difference d after correction of factor J (Hedges’ g = d × J). Thus Hedges’ g (hereinafter referred to as ES) was ultimately used to calculate the effect size in the present study due to the small number of studies for each indicator. According to Cohen’s guiding principle, a small effect was defined as 0.2, a moderate effect as 0.5, and a large effect as 0.8 (Cohen, 1992). Additionally, the I^2^ and *p* of the heterogeneity test are typically used to determine heterogeneity, with I^2^ < 25%, 25% > I^2^ > 50%, and I^2^ > 75% typically used to denote low, moderate, and high heterogeneities (Higgins et al., 2003). However, the *p*-value is also significant because heterogeneity cannot be quantified by I^2^ alone. It is important to consider any potential heterogeneity between studies. Due to the interventions in the various studies included in this analysis, the intervention periods and characteristics were diverse. Therefore, this study applied the random-effects model.

A sensitivity analysis was also performed to confirm the reliability of the results of the meta-analysis. First, whether the effect size of each study fell within the 95% confidence interval of the effect size of the meta-analysis was assessed after omitting one study. The forest map of the sensitivity analysis was then examined to determine whether any study’s effect sizes differed significantly from the total effect size after deleting any study. The results of the meta-analysis were considered reliable if the effect size of all studies fell within the 95% confidence interval of the meta-analysis’s effect size, and there were no discernible differences between the effect size and the total effect size after deleting any study.

## 3 Results

### 3.1 Description of the included studies

**FIGURE 1:**
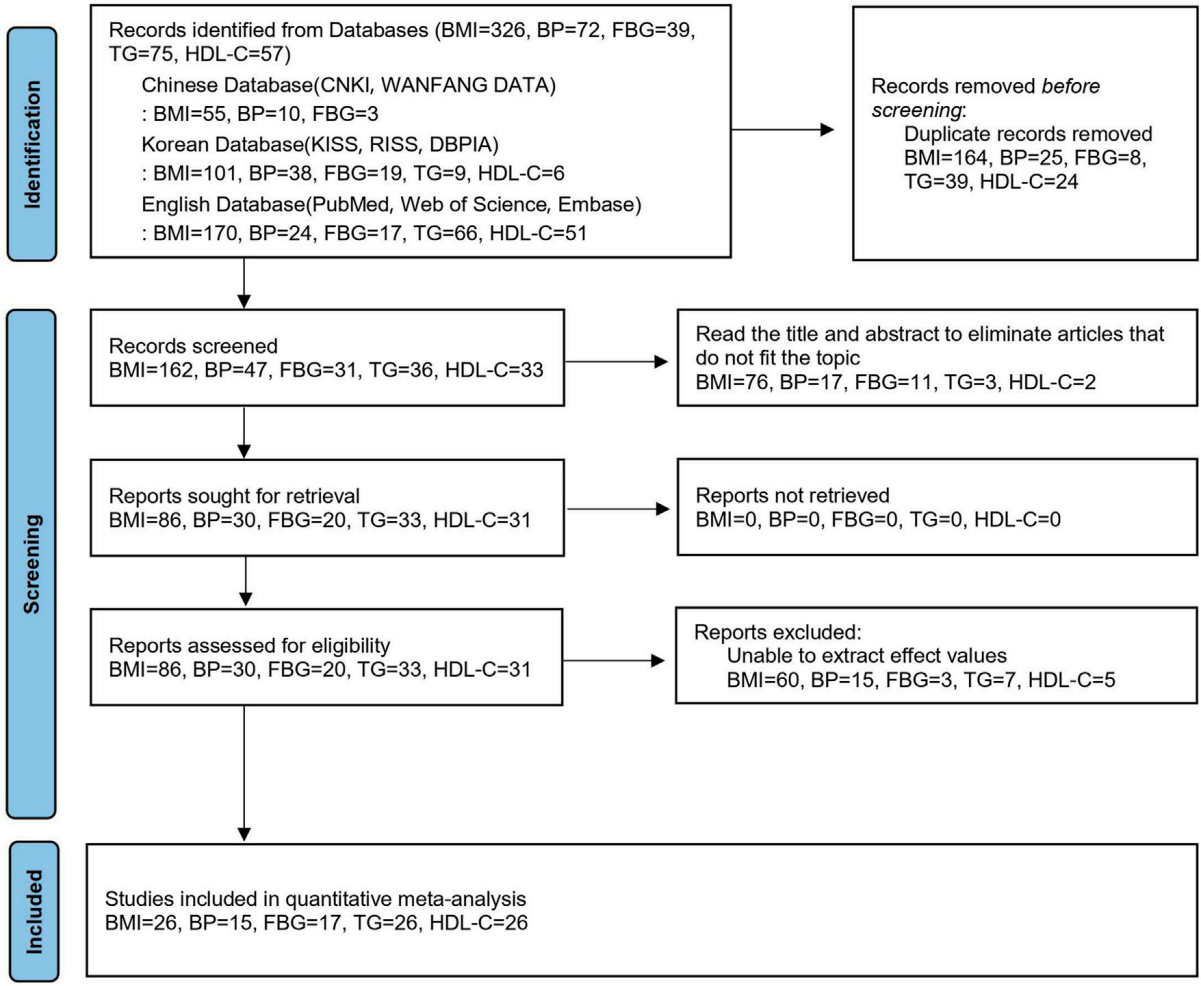
PRISMA 2020 flow diagram.

As illustrated in Figure 1, all retrieved studies were loaded into screening software Zotero 6.0. After screening, a total of 112 studies were included: 1 Chinese, 12 English, and 99 Korean studies. As numerous indicators were retrieved from a single study and one study included two intervention groups (Kim and Yang, 2017), studies containing multiple indicators were combined, resulting in 45 studies (112 effect value), including one Chinese study (Luo, 2017), 6 English studies (Kim, 2015; Jung et al., 2016; Roh et al., 2020; Baek et al., 2021; Kim et al., 2021; Lee et al., 2021), and 38 Korean studies (Choi, 2000; Han et al., 2007; Kang, 2007; 2009; OH and Kim, 2007; Byeon et al., 2008; Kim and Kim, 2008; Kim Y H, 2009a; 2010; 2016; 2021; Kim H. T, 2009; Park et al., 2009; 2010; Ryoo et al., 2009; Ko, 2010; Kwon et al., 2010; 2011a; 2011b; Lee, 2010; 2020; Shin, 2010; Nam et al., 2012; 2013; Cho and Jeoung, 2013; Jang et al., 2013; Cho and Yoon, 2014; Jeong et al., 2014; Lee and Choi, 2014; Kim and Yang, 2017; Lee et al., 2017; 2018; 2020; Kim and Lee, 2018; Koh et al., 2018; Ha et al., 2020; Jeong, 2020; Lee and Kim, 2021). A total of 1079 subjects were included among the studies, including 539 and 540 subjects in the control and intervention groups, respectively. The shortest intervention period was 8 weeks (Kim, 2015; Lee et al., 2017), while the longest cycle was 24 weeks (Lee and Choi, 2014). The included studies were coded according to the following characteristics: first author and year of publication, number of research subjects, sex, age groups, abnormal indicators, intervention cycle, weekly intervention frequency, duration of single interventions, type of intervention, exercise intensity, and final extraction indicators. Table 1 shows the fundamental features of the included studies. According to the features reported in the collected literature, the BMI indicator in this study was divided into an overweight or obese group and a normal group. The International Diabetes Federation’s MS criteria were used to identify whether indicators like BP, FBG, TG, and HDL-C are problematic. The criteria were as follows: BP ≥ 130/85 mmHg, FBG ≥ 100 mg/dl, TG > 150 mg/dl, male HDL-C < 40 mg/dl, and female HDL-C < 50 mg/dl. The intervention types were poomsae, kick, and taekwondo gymnastics. The intervention period was divided into three categories: <12 weeks, 12 weeks, and >12 weeks. The duration of a single intervention was divided into three categories: 40–50 min, 60 min, and 70–90 min. In addition, regarding the classification of exercise intensity, since the indicators reported in the original literature differed, including the three types of maximum heart rate ratio, %HRmax, reserve heart rate ratio %HRR, and maximum oxygen uptake ratio %VO_2_max, the exercise intensity standards were classified as described by Garber et al. For %VO_2_max, <45, 46–63, and 64–90 indicated low, moderate, and high intensities, respectively. For %HRR, 30–39, 40–59, and 60–89 indicated low, moderate, and high intensities, respectively. For %HRmax, <63, 64–76, and 77–95 indicated low, moderate, and high intensities, respectively (Garber et al., 2011).

**TABLE 1:** Basic characteristics of the included studies.

First author (published year)	Number of research subjects	Sex	Age group	Abnormal indicator	Period	Single duration/min	Frequency (times/per week)	Type	Intensity	Final extraction indicator
Luo (2017)	40		Children and teenagers		More than 12 weeks	70–90	3	①+②		①
Lee et al. (2021)	24	Female	Quinquagenarian	①④	More than 12 weeks	60	5	②+③	Moderate	①②④⑤
Kim (2021)	24	Female	Quinquagenarian	①②④⑤	12 weeks	70–90	3	①+③	Moderate	①④⑤
Roh et al. (2020)	20		Children and teenagers	①	More than 12 weeks	60	5	①+②+③		①
Jung et al. (2016)	23	Male	Children and teenagers	①	More than 12 weeks	60	3	①	High	①②
Kim (2015)	14		Youth		Under 12 weeks	70–90	5	②	High	①
Lee et al. (2017)	14	Male	Quinquagenarian	①④	Under 12 weeks	60	3	①+②	Moderate	①④⑤
Lee et al. (2021)	20	Male	Youth	①②③	12 weeks	60	3	①	Moderate	①②③⑤
Kang (2007)	71	Female	Children and teenagers	①	12 weeks	60	4	①+③	Moderate	①②④⑤
Koh et al. (2018)	27		Children and teenagers		12 weeks	40–50	5	①	High	①
Byeon et al. (2008)	30	Female	Quinquagenarian		12 weeks	60	5	①	Moderate	①
Ha et al. (2020)	33	Female	Quinquagenarian	①	More than 12 weeks	60	5	①+②+③	Moderate	①
Jeong (2020)	16	Female	Youth	①④⑤	12 weeks	60	3	①+②	Moderate	①②③④⑤
Lee and Choi (2014)	22		Children and teenagers		More than 12 weeks	60	5	①+②		①
Kim and Kim (2008)	24	Male	Children and teenagers		12 weeks	70–90	5	①+③	High	①
Jang et al. (2013)	16	Male	Children and teenagers	①③	12 weeks	60	5	①+③	Moderate	①②③⑤
Kwon et al. (2010)	24		Children and teenagers	①	12 weeks	60	3	①	Small	①②③④⑤
Jeong et al. (2014)	16	Female	Children and teenagers	①⑤	12 weeks	60	3	①+②+③	Moderate	①②④⑤
Park et al. (2010)	20	Male	Children and teenagers	①	12 weeks	40–50	5	①+②	High	①②③
Choi (2000)	18		Children and teenagers	①	More than 12 weeks	60	3	①+②+③		①②
Cho and Jeoung (2013)	24	Male	Children and teenagers	①⑤	12 weeks	60	3	①	Moderate	①②④⑤
Cho and Yoon (2014)	24	Female	Children and teenagers		12 weeks	60	3	①	Moderate	④
Ryoo et al. (2009)	61		Children and teenagers	③	More than 12 weeks	60	3	①+②		①②③④⑤
Lee et al. (2018)	26	Female	Quinquagenarian	①②	12 weeks	60	3	①+②	High	①②④⑤
Lee et al. (2020)	16		Children and teenagers	①	12 weeks	60	5	①+②+③	Moderate	①②
Baek et al. (2021)	24	Female	Quinquagenarian	②	12 weeks	60	3	①+③	High	②
Park et al. (2009)	30	Male	Children and teenagers	③	12 weeks	60	5	①+②+③	High	①②③④⑤
Kwon et al. (2011a)	16	Female	Quinquagenarian	②③⑤	12 weeks	40–50	3	①	Moderate	②③⑤
Kwon et al. (2011b)	20	Female	Quinquagenarian	②③	12 weeks	40–50	4	①	Moderate	②③
Lee, (2010)	39	Female	Children and teenagers		12 weeks	60	3	①	Moderate	③④
Kim (2021)	17	Female	Youth		Under 12 weeks	60	4	①+②	High	③④⑤
Nam et al. (2013)	20	Female	Youth		12 weeks	60	3	①	Moderate	④⑤
Nam et al. (2012)	28	Female	Youth		12 weeks	60	3	①	Moderate	④⑤
Kim (2016)	20	Female	Quinquagenarian		12 weeks	60	3	①	Moderate	④⑤
Oh and Kim (2007)	14	Female	Children and teenagers		12 weeks	40–50	3	①	Moderate	④⑤
Kim and Yang (2017)	20		Children and teenagers		12 weeks	40–50	5	①+②+③	Moderate	④⑤
Kim and Yang (2017)	20		Children and teenagers		12 weeks	40–50	5	①+②+③	Moderate	④⑤
Kim Y. H (2009)	28	Female	Youth	⑤	12 weeks	60	5	①+②+③	Moderate	④⑤
Lee et al. (2020)	24	Female	Quinquagenarian	⑤	12 weeks	40–50	5	①	High	④⑤
Kim (2010)	20	Male	Youth	④	12 weeks	60	3	①	Moderate	④⑤
Shin (2010)	16	Female	Quinquagenarian	①④⑤	12 weeks	60	3	①	High	①③④⑤
Han et al. (2007)	13	Female	Quinquagenarian	⑤	More than 12 weeks	60	5	①+②	Moderate	③④⑤
Kim and Lee (2018)	14	Male	Youth		12 weeks	60	3	①	Moderate	③
Ko (2010)	20		Children and teenagers		12 weeks	60	5	①+②+③	Small	③④⑤
Kang (2009)	16	Male	Children and teenagers		12 weeks	60	5	①+②+③	High	③④
Kim H. T (2009)	13	Male	Children and teenagers		Under 12 weeks	60	5	①+②	High	③

Note: Abnormal indicators: ①: overweight or obese; ②: abnormal blood pressure; ③: abnormal blood glucose; ④: abnormal triglycerides; ⑤: abnormal high-density lipoprotein cholesterol. Intervention type: ①: poomsae; ②: kick; ③: taekwondo gymnastics. Extraction indicators: ①: BMI; ②: BP; ③: FBG; ④: TG; ⑤: HDL-C. Blank cells: not reported in the original article.

### 3.2 Risk of bias assessment

**FIGURE 2:**
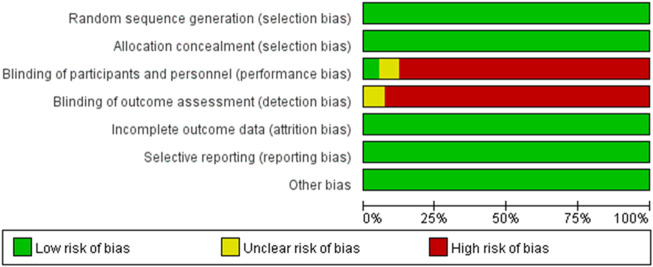
Judgment of bias risk.

The results of the risk of bias assessment are shown in Figure 2. Most studies had high risks of implementation and measurement biases because only two of the studies applied single blinding, while the other studies were not blinded or reported. This situation might have occurred because such studies generally have lengthy interventions, some of the study subjects are minors, and challenges in using a blinded approach because the guardians of these participants must be told in detail about the experimental situation. The other indicators showed modest risk.

### 3.3 Main effect analysis

Table 2 displays the results of the heterogeneity test. The random-effects model was used to combine effect size since BMI, SBP, FBG, TG, HDL-C, and DBP were all moderately (I^2^ > %25, *p* < 0.05) and highly (I^2^ > %75, *p* < 0.001) heterogeneous, respectively. The findings demonstrated that each MS indicator significantly improved because of the taekwondo intervention (Table 2).

**TABLE 2:** Main effects of the indicators of metabolic syndrome.

MS indicator	Number of articles	Number of research subjects	ES	95%CI		Heterogeneity test	
				Lower limit	Upper limit	I^2^	*p*
BMI	26	669	−0.615	−0.754	−0.448	50.832	<0.01
SBP	15	399	−0.672	−0.973	−0.372	52.444	<0.01
DBP	15	399	−0.497	−0.954	−0.039	79.414	<0.001
FBG	17	370	−0.785	−1.079	−0.490	46.782	<0.05
TG	27	661	−0.591	−0.793	−0.390	39.137	<0.05
HDL-C	27	638	0.435	0.223	0.647	44.148	<0.01

### 3.4 Sensitivity analysis

**FIGURE 3:**
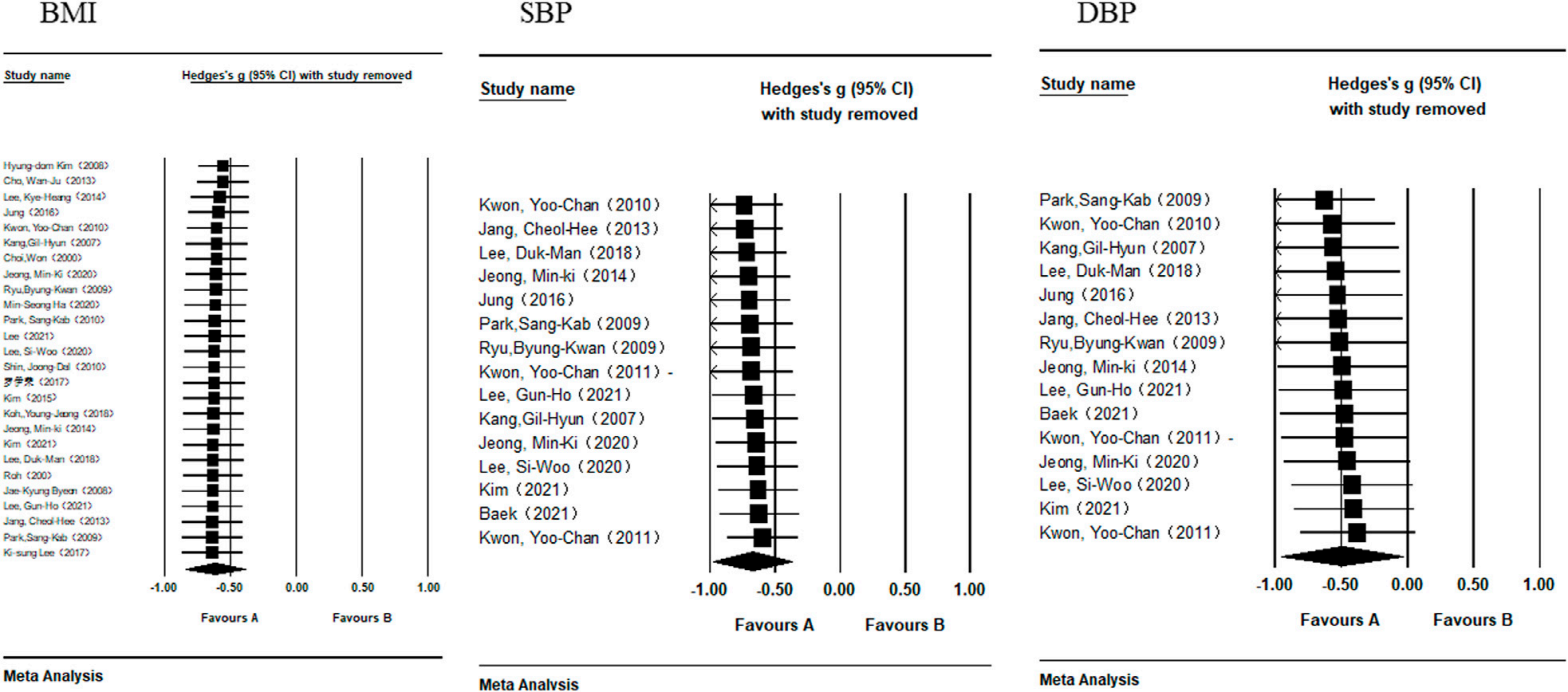
Forest map of the sensitivity analysis of BMI, SBP, and DBP.

**FIGURE 4:**
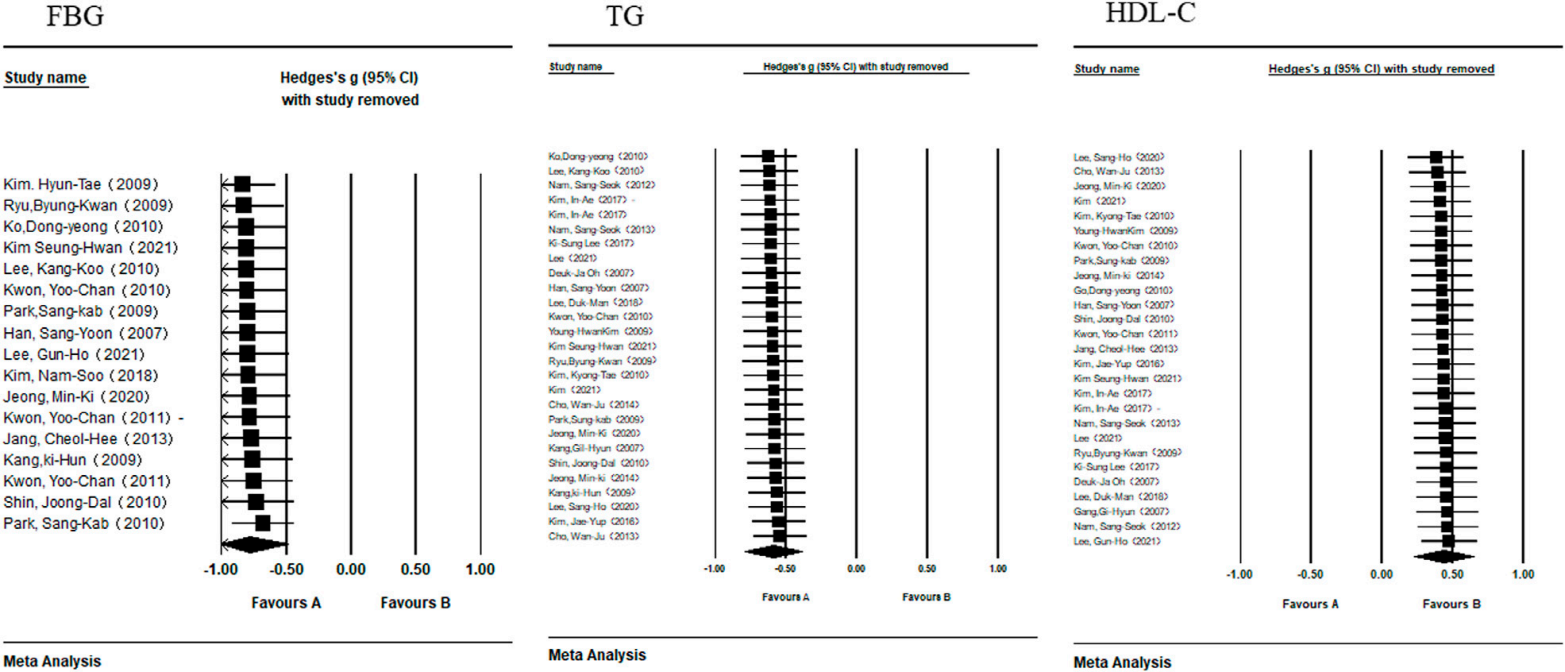
Forest map of the sensitivity analysis of FBG, TG, and HDL-C.

Table 3 displays the results of the sensitivity analysis for each indicator. The effect size of each study after they had been eliminated one by one was all within the 95% confidence interval of the meta-analysis’s effect size. Moreover, the forest map of the sensitivity analysis (Figures 3, 4) showed no significant difference between the effect size and the total effect size after the elimination of individual studies. Therefore, the taekwondo intervention is reliable for the results of the meta-analysis of MS indicators.

**TABLE 3:** Results of the sensitivity analysis.

MS indicator	Number of articles	Number of research subjects	ES	95%CI	ES interval after exclusion one by one
BMI	26	669	−0.615	[−0.839, −0.391]	[−0.553, −0.639]
SBP	15	399	−0.672	[−0.973,-0.372]	[−0.736, −0.598]
DBP	15	399	−0.497	[−0.954, −0.039]	[−0.632, −0.376]
FBG	17	370	−0.785	[−1.079,-0.490]	[−0.839, −0.678]
TG	27	661	−0.591	[−0.793, −0.390]	[−0.623, −0.544]
HDL-C	27	638	0.435	[0.223,0.647]	[0.382, 0.474]

### 3.5 Test of publication bias

**FIGURE 5:**
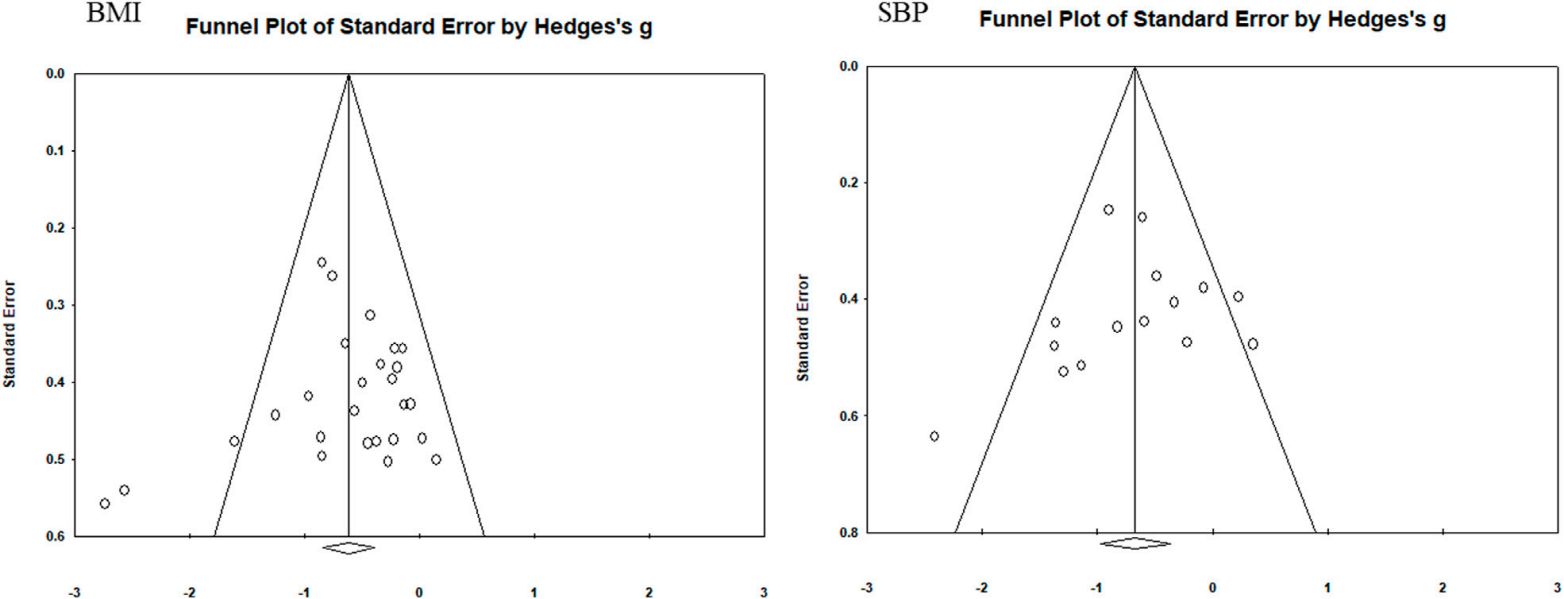
Funnel diagram of BMI and SBP.

**FIGURE 6:**
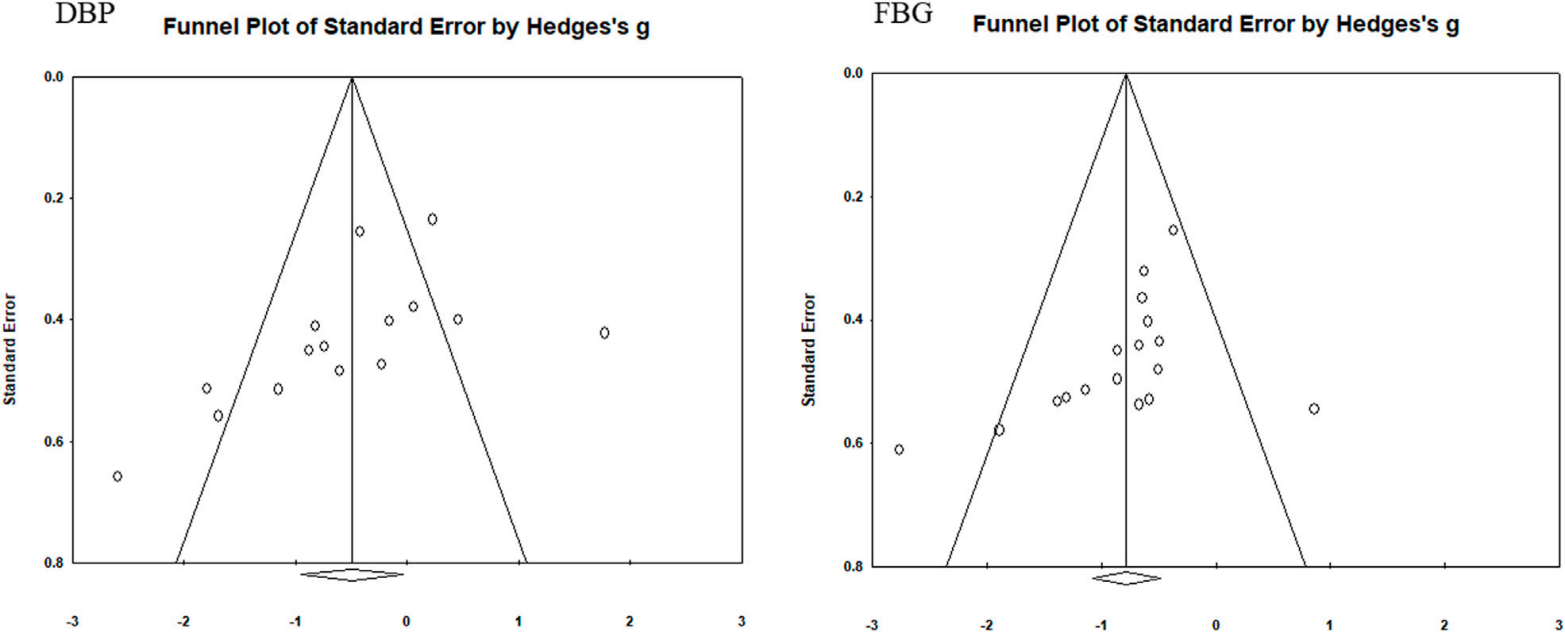
Funnel diagram of DBP and FBG.

**FIGURE 7:**
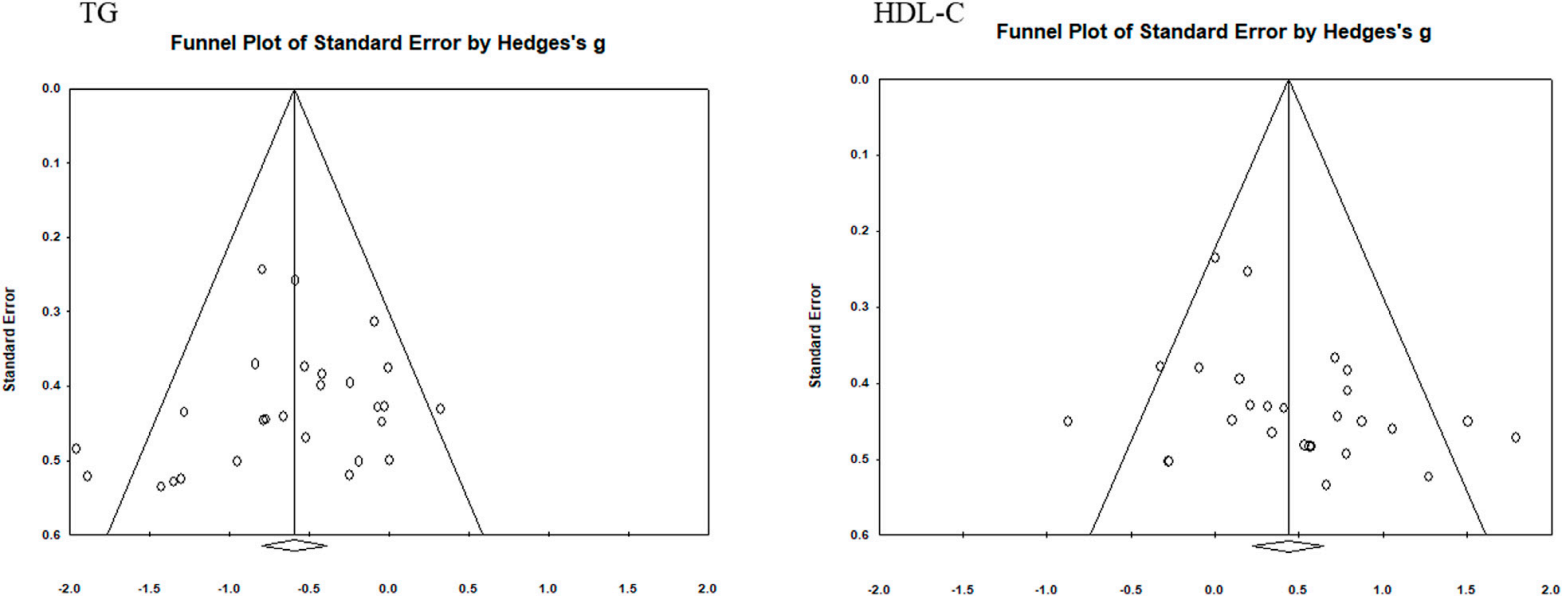
Funnel diagram of TG and HDL-C.

Funnel plots were used to assess the publication bias of each indicator (Figures 5–7). Each funnel plot showed good symmetry. The results of the Egger’s tests showed *p*-values >0.05 for all indicators, indicating no significant publication bias.

### 3.6 Subgroup analysis

This study divided the characteristics of the research subjects and interventions into eight regulatory variables to determine the best intervention strategy. Subgroup analyses were performed according to the following variables: sex, age, MS indicator characteristics, intervention period, single intervention duration, weekly intervention frequency, intervention type, and exercise intensity. To guarantee the representativeness of each subgroup, there should, in theory, be a minimum of three studies per subgroup (Song et al., 2014).

#### 3.6.1 Subgroup analysis of the effect of taekwondo on BMI

The results of the BMI subgroup analysis are shown in Table 4. According to sex, the intervention effect of taekwondo on BMI was greater in men (ES = −0.86, *p* < 0.05) than in women (ES = −0.51, *p* < 0.001). According to age group, the intervention effect in children and adolescents (ES = −0.81, *p* < 0.001) was greater than that in quinquagenarians (ES = −0.32, *p* < 0.05) and was not significant in young people (*p* > 0.05). Regarding BMI, the intervention effect (ES = −0.57, *p* < 0.001) was smaller than normal (ES = −0.73, *p* < 0.05) in overweight or obese people. Regarding intervention cycle, the effects were strong for 12 weeks (ES = −0.62, *p* < 0.001) and more than 12 weeks (−0.72, *p* < 0.001), indicating that the intervention effect increased with increased intervention cycles. Regarding intervention duration, the intervention effect of 60 min per session showed a moderate effect (ES = −0.59, *p* < 0.001), while the intervention effect of 70–90 min per time was not significant (*p* > 0.05). Regarding intervention frequency, the effect of interventions performed three times a week (ES = −0.63, *p* < 0.001) was greater than that of five times a week (ES = −0.58, *p* < 0.05). Regarding intervention type, the intervention effect of poomsae was the best (ES = −0.77, *p* < 0.05), followed by poomsae + kick (ES = −0.60, *p* < 0.001) and poomsae + kick + taekwondo gymnastics (ES = −0.40, *p* < 0.05). The intervention effect of poomsae + taekwondo gymnastics was not significant (*p* > 0.05). The intervention effect of moderate-intensity interventions (ES = −0.52, *p* < 0.05) was lower than that of high-intensity interventions (ES = −0.68, *p* < 0.05).

**TABLE 4:** BMI subgroup analysis.

Regulated variable	Subgroup	Number of articles	Heterogeneity (subgroup)		Regulatory effect	ES	Two-tailed test	
			I^2^ (%)	*p*	*p*		Z	*p*
Sex	Male	8	80.87	<0.001	>0.05	−0.86	−2.31	<0.05
	Female	9	0.00	>0.05		−0.51	−4.09	<0.001
Age group	Children and teenagers	16	63.07	<0.001	>0.05	−0.81	−4.88	<0.001
	Youth	3	0.00	>0.05		−0.37	−1.34	>0.05
	Quinquagenarian	7	0.00	>0.05		−0.32	−2.10	<0.05
BMI feature	Overweight or obesity	18	37.70	>0.05	>0.05	−0.57	−4.53	<0.001
	Normal	8	70.17	<0.001		−0.73	−2.96	<0.05
Intervention cycle	12 weeks	16	61.85	<0.001	>0.05	−0.62	−3.66	<0.001
	More than 12 weeks	8	13.59	>0.05		−0.72	−5.04	<0.001
	Under 12 weeks	2						
Single intervention duration	70–90 min	4	81.41	<0.05	>0.05	−0.86	−1.72	>0.05
	60 min	22	39.32	<0.05		−0.59	−5.27	<0.001
Frequency of weekly intervention	Three times	13	49.61	<0.05	>0.05	−0.63	−3.87	<0.001
	Five times	12	57.08	<0.05		−0.58	−3.09	<0.05
	Four times	1						
Type of intervention	①	7	68.75	<0.05	>0.05	−0.77	−2.67	<0.05
	①+②	7	31.23	>0.05		−0.60	−3.36	<0.001
	①+②+③	6	0.00	>0.05		−0.40	−2.36	<0.05
	①+③	4	82.50	<0.05		−0.89	−1.88	>0.05
	②	1						
	②+③	1						
Exercise intensity	Small	1						
	Moderate	12	50.98	<0.05	>0.05	−0.52	−3.04	<0.05
	High	8	66.27	<0.05		−0.68	−2.56	<0.05

Note: ①: poomsae; ②: kick; ③: taekwondo gymnastics.

#### 3.6.2 Subgroup analysis of the effect of taekwondo on SBP

The results of the SBP subgroup analysis are shown in Table 5. According to sex, the intervention effect of taekwondo on SBP was not significant in men (*p* > 0.05) but was large in women (ES = −0.92, *p* < 0.001). According to age group, the intervention effect in quinquagenarians (ES = −1.08, *p* < 0.05) was much larger than that in children and adolescents (ES = −0.45, *p* < 0.05). Regarding SBP, the intervention effect in the abnormal population (ES = −1.02, *p* < 0.05) was much greater than that in the normal population (ES = −0.5, *p* < 0.05). Regarding intervention frequencies, three times a week showed a moderate effect (ES = −0.73, *p* < 0.001), while five times a week showed no significant effect (*p* > 0.05). Regarding intervention types, poomsae + taekwondo gymnastics showed the best effect (ES = −0.83, *p* < 0.05), followed by poomsae + kick + taekwondo gymnastics (ES = −0.61, *p* < 0.05) and poomsae + kick (ES = −0.55, *p* < 0.05). The intervention effect of poomsae was not significant (*p* > 0.05). The intervention effect of moderate intensity intervention (ES = −0.87, *p* < 0.001) was higher than that of high intensity (ES = −0.53, *p* < 0.05).

**TABLE 5:** SBP subgroup analysis.

Regulated variable	Subgroup	Number of articles	Heterogeneity (subgroup)		Regulatory effect	ES	Two-tailed test	
			I^2^ (%)	*p*	*p*		Z	*p*
Sex	Male	3	42.05	>0.05	>0.05	−0.35	−1.08	>0.05
	Female	8	53.71	<0.05		−0.92	−4.15	<0.001
Age group	Children and teenagers	8	42.65	>0.05	>0.05	−0.45	−2.58	<0.05
	Quinquagenarian	5	68.86	<0.05		−1.08	−2.90	<0.05
	Youth	2						
SBP feature	Abnormal	6	61.23	<0.05	>0.05	−1.02	−3.37	<0.05
	Normal	9	41.39	>0.05		−0.50	−3.00	<0.05
Frequency of weekly	Three times	10	59.55	<0.05	>0.05	−0.73	−3.43	<0.001
Intervention	Five times	3	63.21	>0.05		−0.45	−1.06	>0.05
	Four times	2						
Type of intervention	①	5	69.45	<0.05	>0.05	−0.69	−1.87	>0.05
	①+②	3	31.28	>0.05		−0.55	−2.16	<0.05
	①+②+③	3	19.24	>0.05		−0.61	−2.14	<0.05
	①+③	4	66.55	<0.05		−0.83	−2.42	<0.05
Exercise intensity	Small	1						
	Moderate	9	52.32	<0.05	>0.05	−0.87	−4.02	<0.001
	High	4	42.71	>0.05		−0.53	−2.05	<0.05

Note: ①: poomsae; ②: kick; ③: taekwondo gymnastics.

#### 3.6.3 Subgroup analysis of the effect of taekwondo on DBP

The results of the DBP subgroup analysis are shown in Table 6. Among the sexes, the intervention effect showed no significance in men (*p* > 0.05), while a large effect was observed in women (ES = −0.88, *p* < 0.05). According to age groups, the intervention effect was not significant in children and adolescents (*p* > 0.05) but showed a large effect in quinquagenarians (ES = −1.16, *p* < 0.05). Regarding DBP, the intervention effect was large for abnormal DBP (ES = −1.03, *p* < 0.05) and not significant for normal DBP (*p* > 0.05). Regarding intervention frequencies, three times a week showed a moderate effect (ES = −0.67, *p* < 0.001), while five times a week showed no significant effect (*p* > 0.05). No intervention type showed a significant intervention effect (*p* > 0.05). Regarding exercise intensities, the intervention effect was large for moderate intensity (ES = −0.96, *p* < 0.001) but not significant for high intensity (*p* > 0.05).

**TABLE 6:** DBP subgroup analysis.

Regulated variable	Subgroup	Number of articles	Heterogeneity (subgroup)		Regulatory effect	ES	Two-tailed test	
			I^2^ (%)	*p*	*p*		Z	*p*
Sex	Male	3	89.31	<0.001	>0.05	0.28	0.34	>0.05
	Female	8	77.89	<0.001		−0.88	−2.65	<0.05
Age group	Children and teenagers	8	79.16	<0.001	<0.05	−0.04	−0.14	>0.05
	Quinquagenarian	5	75.42	<0.05		−1.16	−2.63	<0.05
	Youth	2						
DBP feature	Abnormal	6	69.05	<0.05	<0.05	−1.03	−3.02	<0.05
	Normal	9	79.18	<0.001		−0.15	−0.53	>0.05
Frequency of weekly	Three times	10	67.42	<0.001	>0.05	−0.67	−2.86	<0.001
Intervention	Five times	3	92.30	<0.001		−0.02	−0.02	>0.05
	Four times	2						
Type of intervention	①	5	77.21	<0.05	>0.05	−0.68	−1.59	>0.05
	①+②	3	45.10	>0.05		−0.43	−1.48	>0.05
	①+②+③	3	92.84	<0.001		−0.15	−0.14	>0.05
	①+③	4	80.09	<0.05		−0.59	−1.34	>0.05
Exercise intensity	Small	1						
	Moderate	9	75.69	<0.001	>0.05	−0.96	−3.15	<0.001
	High	4	85.63	<0.001		0.21	0.40	>0.05

Note: ①: poomsae; ②: kick; ③: taekwondo gymnastics.

#### 3.6.4 Subgroup analysis of the effect of taekwondo on FBG

The results of the FBG subgroup analysis are shown in Table 7. Among the sexes, the intervention effect was slightly lower in men (ES = −0.65, *p* < 0.001) than in women (ES = 0.68, *p* < 0.001). According to age group, the intervention effect was large in children and adolescents (ES = −0.9, *p* < 0.001) but was not significant in quinquagenarians (*p* > 0.05). Regarding FBG, the intervention effect was slightly higher in the abnormal population (ES = −0.85, *p* < 0.001) than in the normal population (ES = −0.76, *p* < 0.001). Regarding intervention duration, the effect of a 40–50 min intervention (ES = −0.85, *p* < 0.05) was slightly greater than that of a 60 min intervention (ES = −0.77, *p* < 0.001). The effect of interventions performed five times a week (ES = -0.91, *p* < 0.05) was greater than that of interventions three times a week (ES = -0.67, *p* < 0.001). Among different intervention types, the intervention effect of poomsae + kick + taekwondo gymnastics (ES = −1.69, *p* < 0.05) was significantly higher than that of poomsae (ES = −0.67, *p* < 0.001). The intervention effect of poomsae + kick was not significant (*p* > 0.05). The effect of high-intensity exercise (ES = -0.81, *p* < 0.001) was significantly higher than that of moderate-intensity exercise (ES = −0.58, *p* < 0.001).

**TABLE 7:** FBG subgroup analysis.

Regulated variable	Subgroup	Number of articles	Heterogeneity (subgroup)		Regulatory effect	ES	Two-tailed test	
			I^2^ (%)	*p*	*p*		Z	*p*
Sex	Male	7	13.01	>0.05	>0.05	−0.65	−4.31	<0.001
	Female	7	48.49	>0.05		−0.68	−3.91	<0.001
Age group	Children and teenagers	10	42.82	>0.05	>0.05	−0.90	−4.72	<0.001
	Quinquagenarian	5	65.19	<0.05		−0.65	−1.69	>0.05
	Youth	2						
FBG feature	Abnormal	6	0.00	>0.05	>0.05	−0.85	−4.51	<0.001
	Normal	11	63.59	<0.05		−0.76	−3.43	<0.001
Single intervention	60 min	14	53.72	<0.05	>0.05	−0.77	−4.39	<0.001
Duration	40–50 min	3	0.00	>0.05		−0.85	−3.16	<0.05
Frequency of weekly	Three times	8	0.00	>0.05	>0.05	−0.67	−4.78	<0.001
Intervention	Five times	7	75.46	<0.001		−0.91	−2.39	<0.05
	Four times	2						
Type of intervention	①	7	0.00	>0.05	>0.05	−0.67	−4.68	<0.001
	①+②	6	25.14	>0.05		−0.40	−1.67	>0.05
	①+②+③	3	79.94	<0.05		−1.69	-2.56	<0.05
	①+③	1						
Exercise intensity	Small	2						
	High	6	12.57	>0.05	>0.05	−0.81	−4.21	<0.001
	Moderate	8	41.53	>0.05		−0.58	−4.12	<0.001

Note: ①: poomsae; ②: kick; ③: taekwondo gymnastics.

#### 3.6.5 Subgroup analysis of the effect of taekwondo on TG

The results of the TG subgroup analysis are shown in Table 8. Among the sexes, the intervention effect was slightly lower in men (ES = −0.61, *p* < 0.05) than in women (ES = −0.65, *p* < 0.001). According to age group, the intervention effect was greatest in quinquagenarian (ES = −0.81, *p* < 0.001), followed by children and adolescents (ES = −0.54, *p* < 0.001) and young subjects (ES = −0.41, *p* < 0.05). Regarding TG, the intervention effect in the abnormal population (ES = -0.72, *p* < 0.001) was greater than that in the normal population (ES = -0.57, *p* < 0.001). Regarding intervention cycles, the effect of 12 weeks (ES = −0.65, *p* < 0.001) was greater than that of more than 12 weeks (ES = −0.45, *p* < 0.05). Regarding intervention duration, a single intervention of 70–90 min showed a moderate effect (ES = −0.66, *p* < 0.001), while a single intervention of 60 min showed no significant effect (*p* > 0.05). Regarding intervention frequencies, three times a week had a moderate effect (ES = −0.69, *p* < 0.001), while five times a week showed no significant effect (*p* > 0.05). Regarding intervention types, poomsae showed the best effect (ES = −0.78, *p* < 0.001), followed by poomsae + kick + taekwondo gymnastics (ES = −0.53, *p* < 0.05) and poomsae + kick (ES = −0.39, *p* < 0.05). The effect of high-intensity exercise (ES = −0.74 (*p* < 0.001) was more significant than that of moderate-intensity exercise (ES = −0.55 (*p* < 0.001).

**TABLE 8:** TG subgroup analysis.

Regulated variable	Subgroup	Number of articles	Heterogeneity (subgroup)		Regulatory effect	ES	Two-tailed test	
			I^2^ (%)	*p*	*p*		Z	*p*
Sex	Male	7	59.03	<0.05	>0.05	−0.61	−2.38	<0.05
	Female	17	35.43	>0.05		−0.65	−5.05	<0.001
Age group	Children and teenagers	12	50.35	<0.05	>0.05	−0.54	−3.45	<0.001
	Youth	6	0.00	>0.05		−0.41	−2.37	<0.05
	Quinquagenarian	9	44.76	>0.05		−0.81	−3.84	<0.001
TG feature	Abnormal	5	0.00	>0.05	>0.05	−0.72	−3.37	<0.001
	Normal	22	45.68	<0.05		−0.57	−4.85	<0.001
Intervention cycle	12 weeks	22	47.46	<0.05	>0.05	−0.65	−5.27	<0.001
	More than 12 weeks	3	0.00	>0.05		−0.45	−2.24	<0.05
	Under 12 weeks	2						
Single intervention	70–90 min	21	36.46	<0.05	>0.05	−0.66	−5.84	<0.001
Duration	60 min	5	48.96	>0.05		−0.25	−0.89	>0.05
	40–50 min	1						
Frequency of weekly	Three times	17	46.62	<0.05	>0.05	−0.69	−4.81	<0.001
Intervention	Five times	8	29.95	>0.05		−0.38	−2.15	>0.05
	Four times	2						
Type of intervention	①	12	60.30	<0.001	>0.05	−0.78	−3.86	<0.001
	①+②	7	0.00	>0.05		−0.39	−2.57	<0.05
	①+②+③	5	28.30	>0.05		−0.53	−2.39	<0.05
	①+③	2						
	②+③	1						
Exercise intensity	Small	1						
	High	7	47.16	>0.05	>0.05	−0.74	−3.21	<0.001
	Moderate	18	44.03	<0.05		−0.55	−4.13	<0.001

Note: ①: poomsae; ②: kick; ③: taekwondo gymnastics.

#### 3.6.6 Subgroup analysis of the effect of taekwondo on HDL-C

The results of the HDL-C subgroup analysis are shown in Table 9. Among the sexes, no significant intervention effect was observed in men (*p* > 0.05), while the intervention effect in females was moderate (ES = 0.53, *p* < 0.001). According to age group, the intervention effect in quinquagenarian (ES = 0.65, *p* < 0.05) was greater than that in children and adolescents (ES = 0.33, *p* < 0.05), with no significant difference in the intervention effect in young people (*p* > 0.05). In the HDL-C group, the intervention effect in the abnormal population showed a large effect (ES = 1.05, *p* < 0.001), while the intervention effect in the normal population was not statistically significant (*p* > 0.05). According to intervention duration, a single intervention of 60 min had a moderate effect (ES = 0.45, *p* < 0.001), while the effect of 40–50 min was not statistically significant (*p* > 0.05). Regarding intervention frequencies, the effect of interventions performed five times a week (ES = 0.52, *p* < 0.001) was slightly higher than that for three times a week (ES = 0.48, *p* < 0.05). Among intervention types, the intervention effect of poomsae (ES = 0.5, *p* < 0.05) was lower than that of poomsae + kick + taekwondo gymnastics (ES = 0.62, *p* < 0.05), with no significant effects in other intervention types (*p* > 0.05). The effect of high-intensity interventions (ES = 0.67, *p* < 0.05) was significantly greater than that of moderate-intensity interventions (ES = 0.40, *p* < 0.05).

**TABLE 9:** HDL-C subgroup analysis.

Regulated variable	Subgroup	Number of articles	Heterogeneity (subgroup)		Regulatory effect	ES	Two-tailed test	
			I^2^ (%)	*p*	*p*		Z	*p*
Sex	Male	6	59.83	<0.05	>0.05	0.33	1.12	>0.05
	Female	17	51.11	<0.05		0.53	3.41	<0.001
Age group	Children and teenagers	10	0.00	>0.05	>0.05	0.33	2.76	<0.05
	Youth	7	63.43	<0.05		0.31	1.07	>0.05
	Quinquagenarian	10	52.99	<0.05		0.65	2.96	<0.05
HDL-C feature	Abnormal	9	0.00	>0.05	<0.001	1.05	6.43	<0.001
	Normal	18	11.78	>0.05		0.20	1.92	>0.05
Single intervention	70–90 min	1						
Duration	60 min	21	51.76	<0.05	>0.05	0.45	3.33	<0.001
	40–50 min	5	0.00	>0.05		0.39	1.90	>0.05
Frequency of weekly	Three times	17	60.65	<0.001	>0.05	0.48	2.76	<0.05
Intervention	Five times	8	0.00	>0.05		0.52	3.34	<0.001
	Four times	2						
Type of intervention	①	12	64.44	<0.001	>0.05	0.50	2.18	<0.05
	①+②	5	30.55	>0.05		0.25	1.09	>0.05
	①+②+③	6	0.00	>0.05		0.62	3.43	<0.05
	①+③	3	56.98	>0.05		0.46	1.30	>0.05
	②+③	1						
Exercise intensity	Small	2						
	High	5	60.28	<0.05	>0.05	0.67	2.10	<0.05
	Moderate	19	46.48	<0.05		0.40	2.84	<0.05

Note: ①: poomsae; ②: kick; ③: taekwondo gymnastics.

### 3.7 Maximization of the intervention benefits of each indicator

The intervention effects of each indicator for various intervention characteristics were merged to identify the greatest intervention effect of taekwondo on each indicator after eliminating ineffective subgroups (less than three studies or no statistical significance). This process required two steps. First, whether the intervention frequency, when used in the same intervention cycle, had the best effect on each indicator was assessed. Second, which exercise intensity and intervention type have the best results for each indicator when used for the same duration of effect throughout a single intervention were determined. The results are displayed in Table 10. The effect of taekwondo intervention on BMI was maximized by moderate-intensity poomsae training for 60 min, three times weekly for more than 12 weeks. For SBP, moderate-intensity poomsae + kick + taekwondo gymnastics training for 60 min three times weekly for 12 weeks showed the maximum effect. For DBP, the effect was maximized with moderate-intensity taekwondo training for 60 min three times weekly for 12 weeks. For FBG, high-intensity poomsae + kick + taekwondo gymnastics training for 60 min five times weekly for 12 weeks maximized the effect. For TG, high-intensity poomsae + kick + taekwondo gymnastics for 60 min three times weekly for 12 weeks showed the maximum intervention effect. For HDL-C, moderate-intensity poomsae + Kick + taekwondo gymnastics training for 60 min five times weekly for 12 weeks showed the maximum intervention effect.

**TABLE 10:** Intervention effects of different intervention characteristics.

MS indicator	Period	Frequency (per week)		Single duration	Exercise intensity		Type		
		Three times	Five times		Moderate	High	①	①+②	①+②+③
BMI	12 weeks	−0.639	−0.566	60 min	−0.602	−0.416	−0.803	−0.661	−0.416
	More than 12 weeks	−0.744	−0.687						
SBP	12 weeks	−0.972	—	60 min	−0.816	−0.543	—	−0.561	−0.634
DBP	12 weeks	−0.710	—	60 min	−0.858	—	—	—	—
FBG	12 weeks	−0.670	−1.295	60 min	−0.487	−0.903	−0.599	—	−1.773
TG	12 weeks	−0.782	−0.415	60 min	−0.635	−0.815	−0.827	−0.511	−0.841
HDL-C	12 weeks	0.551	0.560	60 min	0.408	—	—	—	0.751
				40–50 min	0.332	—	—	—	—

“-”, invalid value (*p* > 0.05 or fewer than three studies in the subgroup); ①: poomsae; ②: kick; ③: taekwondo gymnastics.

## 4 Discussion

### 4.1 Sources of heterogeneity

By assessing the heterogeneity between subgroups, this study investigated the source of heterogeneity in all indicators. Male and female heterogeneity in the FBG, sex, and exercise intensity subgroups was not significant (I^2^ = 13.1,48.49; *p* > 0.05), suggesting that sex and exercise intensity were sources of inter-study heterogeneity. Abnormal and normal heterogeneity became insignificant in the subgroup of HDL-C MS indicator characteristics (I^2^ = 0,11.78; *p* > 0.05), indicating that the source of heterogeneity in HDL-C studies may be related to these factors. The other indicators showed no significant subgroup heterogeneity.

### 4.2 Regulating effect

Age and DBP characteristics showed significant regulatory effects (*p* < 0.05), indicating that the intervention effect of taekwondo on DBP may be influenced by age and DBP characteristics. HDL-C characteristics also showed a significant regulatory effect (*p* < 0.05), indicating that the intervention effect of taekwondo on HDL-C may be influenced by HDL-C characteristics. The moderating effects of each variable in the other indicators were not significant.

### 4.3 Main effect

To our knowledge, this study is the first meta-analysis to evaluate the effect of a taekwondo intervention on MS indicators. Our results showed that taekwondo interventions dramatically improved MS indicators. Taekwondo had a positive intervention effect on MS indicators like BMI, TG, and HDL-C (Yang, 2020; Lei et al., 2022). Additionally, a meta-analysis of the effects of MS found that aerobic exercise (Ostman et al., 2017), endurance training (Pattyn et al., 2013), resistance exercise (Wewege et al., 2018), and unsupervised exercise (Peiris et al., 2021) all produced positive results, which supported the results of the present study. However, unsupervised exercise (Peiris et al., 2021) and endurance training (Pattyn et al., 2013) did not significantly affect FBG and HDL-C or improve TG and FBG. Taekwondo may provide superior intervention effects on MS indicators compared to these workouts as taekwondo involves complicated movements that require the synchronization of numerous body parts, such as twisting blocks, flying kicks, and fast motions. Taekwondo is not restricted to just one portion of the body. Taekwondo training also frequently includes training that can improve participant engagement. Additionally, the arrangement of exercise intensity is sometimes unbalanced during this process.

### 4.4 Research subject characteristics

The research subjects in the present study were categorized according to sex, age, and MS characteristics. First, analysis of the effects of taekwondo on MS indicators according to sex showed that women experienced a generally better effect to the intervention compared to men, similar to the results reported by Kubaisy et al. (2015) . Women performed better than men at regular exercise. Women may have higher body image awareness, which makes them more committed to exercising. One study reported that women felt pride when they participated in sports or guilt if they did not (Wilson and Brookfield, 2009). Second, the findings of the present study indicated that taekwondo interventions had greater overall effects on MS indicators in quinquagenarians than in other age groups. A further finding from studies is that muscle mass declines with age (Crane et al., 2013). Therefore, older participants will exert more exercise than the young under for same intensity of taekwondo exercise intervention, producing relatively good exercise effects. The results of this study also showed that taekwondo intervention had a greater effect on people with abnormal MS indicators compared to those with normal MS indicators, considering various MS characteristics. However, in their meta-analysis, Kodama et al. (2007) reported better exercise results in people with normal BMI and HDL-C indicators. This may be because the exercise characteristics in the study by Kodama et al. only considered the exercise duration, frequency, and intensity and overlooked the exercise cycle, which may have resulted in different results. Additionally, Kodama et al.’s study was restricted to regular aerobic exercise, and various types of exercise may also yield various results. The results of the present study indicated that a taekwondo intervention can maximize the benefit in quinquagenarian women with abnormal MS indicator levels.

### 4.5 Intervention characteristics

This study divided the intervention characteristics into intervention time, intervention frequency, exercise intensity, and intervention type. The results showed that a taekwondo intervention lasting for 12 weeks had the best all-around effect on MS indicators after omitting ineffective subgroups (fewer than three studies). Numerous studies reported the necessity for consistent, long-term physical exercise to successfully alter body composition and lower cardiovascular risk factors (Dustman et al., 1984; Hagberg et al., 1989; Ma, 2022). Moreover, an intervention of sprint interval training significantly altered the TG of overweight women after 12 weeks but not after 6 weeks (Alkahtani, 2014). Although the results of these studies were comparable to those of the present study, studies reported contradictory results. Amundsen et al. (1989)and Bakken et al. (2001) showed that 8 weeks of exercise was all necessary to achieve good training results. However, the two studies included elderly intervention subjects, whereas the intervention subjects in the present study spanned all age groups except for infants. This may explain the discordant findings from the present study. The results of the two studies also confirm that the taekwondo intervention was more successful in quinquagenarians.

Regarding intervention frequencies, omitting ineffective subgroups (containing fewer than three studies), the results of the present study showed the highest intervention effect for 40–50 min sessions performed three times. This prescription is close to the recommendation from the American College of Sports Medicine (30 min or more of moderate-intensity physical exercise most days of the week, ideally every day) (Pate et al., 1995). Kodama et al. (2007) also found that more than two sessions per week were required to significantly increase HDL-C levels and that each continuous exercise session must last longer than the duration advised by the American College of Sports Medicine

Our findings showed that high-intensity taekwondo had a greater impact on the other MS indicators except for SBP and DBP. In general, moderate to high aerobic exercise is advised to prevent or reduce cardiovascular risk factors (Arnett et al., 2019; American Diabetes Association, 2020). However, some researchers have suggested that higher levels of exercise intensity tend to have greater positive effects on glycated hemoglobin (HbA1c) level, aerobic capacity, and health (Colberg et al., 2016). The results of the present study generally suggest high-intensity taekwondo training to maximize the intervention effect. However, it is important to consider that recommending more strenuous exercise when the intervention target is an obese or weak patient may result in dissatisfaction and decreased compliance (Schwaab et al., 2020). Thus, the exercise intensity should consider the characteristics of the intervention object and be generally set to medium or high intensity.

**FIGURE 8:**
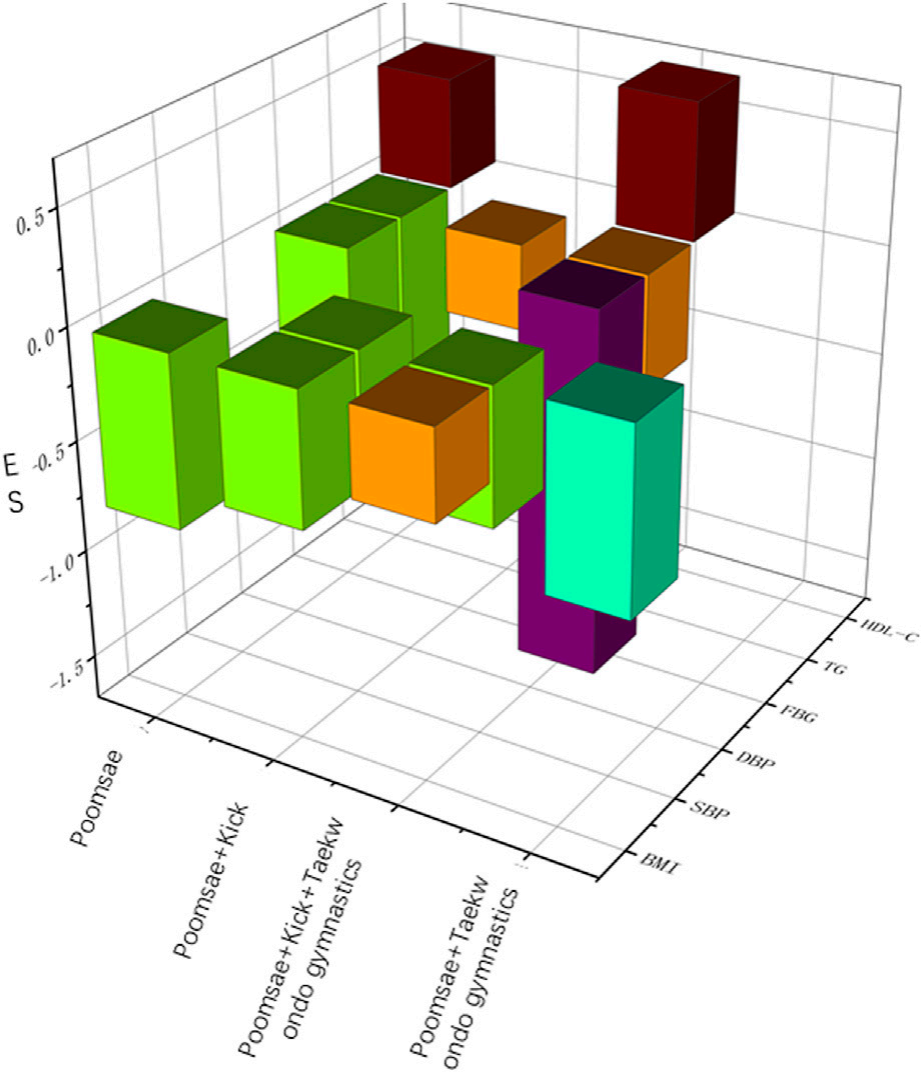
Effect sizes for different intervention types (3D).

Regarding intervention types, it was challenging to determine which type of comprehensive intervention has the best effect from the results after omitting ineffective subgroups (those with fewer than three studies). Thus, a 3D bar chart was created using the intervention effect size of four different intervention types on each MS indicator (Figure 8). Poomsae + kick + taekwondo gymnastics could be roughly extrapolated to have the most extensive intervention effect on MS indicators. A certain type of training in taekwondo may not have a significant impact on various MS indicators when used alone. Lee and Kim (2021)showed that only the taekwondo kick training intervention had a positive effect on BMI and TG, with no significant effects on HDL-C. Lee et al. (2018)reported that poomsae and kick training alone did not significantly improve SBP, DBP, and HDL-C. These early studies helped to validate the results of the present study, suggesting that an intervention combining poomsae + kick + taekwondo gymnastics would be better.

### 4.6 Limitations

1) This study included only studies in Chinese, Korean, and English. However, except for one Chinese study, all the experimental regions were in South Korea and the subjects were predominantly Korean, which limited the generalizability of the findings. 2) An excessive number of invalid subgroups in the subgroup analysis hindered the output of the research results. 3) The indicators of reported exercise intensity differed among the studies. Even if the units were unified through secondary classification, there remained some variations, particularly for some indicators close to the intensity threshold, which may lead to errors in grading and, ultimately, errors in the results.

## 5 Conclusion

The results showed that taekwondo effectively improved MS, as manifested by decreased BMI, SBP, DBP, FBG, and TG levels and increased HDL-C level. To maximize the intervention effect of taekwondo on MS, high-intensity poomsae + kick + taekwondo gymnastics training is recommended as an exercise prescription three times weekly for 40–50 min per session for 12 weeks. In addition, for single indicators, the recommended exercise prescriptions are as follows: 1) BMI, three times weekly for 60 min per session, moderate-intensity poomsae training for >12 weeks; 2) SBP, moderate-intensity poomsae + kick + taekwondo gymnastics training for 12 weeks, three times per week, 60 min per session; 3) DBP, moderate-intensity taekwondo for 60 min three times per week for 12 weeks; 4) FBG, 60 min of high-intensity poomsae + kick + taekwondo gymnastics training five times per week for 12 weeks; 5) TG, high-intensity poomsae + kick + taekwondo gymnastics three times per week, 60 min per session, for 12 weeks; 6) HDL-C, moderate-intensity poomsae + kick + taekwondo gymnastics training for 60 min five times a week for 12 weeks. Given the limitations of this study, more well-designed RCTs and systematic reviews are needed to improve such studies in the future.

## Data Availability

The raw data supporting the conclusion of this article will be made available by the authors, without undue reservation.
