# Supplementary material for: Meta-analysis of the intervention effects of taekwondo on metabolic syndrome indicators
Source: Front Physiol. 2023 Jan 17;14:1069424. doi: 10.3389/fphys.2023.1069424 (PMC9887190; doi:10.3389/fphys.2023.1069424)
Supplement: Supplementary file 1 [file DataSheet1.doc]

Supplementary material：Full search strategies

1. Date of last database search: September 25, 2022. A search was conducted for literature that had been published prior to September 25, 2022.

2. Database

（1）Chinese Database

China National Knowledge Infrastructure（CNKI）：https://cnki.net/

Wanfang Data Knowledge Service Platform（WANFANG DATA）：https://www.wanfangdata.com.cn/

（2）Korean Database

Korean-studies Information Service System（KISS）：https://kiss.kstudy.com/

Research Information Sharing Service（RISS）：http://www.riss.kr/index.do

KRpiaStudies Database（DBPIA）：https://www.dbpia.co.kr/

（3）English Database

PubMed：https://pubmed.ilibs.cn/

Web of Science：https://www.webofscience.com/

Excerpta Medica Database（Embase）：<https://www.embase.com/>

3. Search strategy

（1）Chinese Database

We used the advanced search function to set keyword 1 to "taekwondo" and keyword 2 to "BMI" or "waist circumference" or "metabolic syndrome" or "MS" or "obesity" or "SBP" or "blood pressure" or "DBP" or "fasting glucose" or "FBG" or "TG" or "triglycerides" or "HDL-C" or "HDL cholesterol" for the advanced search.

（2）Korean Database

Using the detailed search function, we set keyword 1 to "taekwondo" and keyword 2 to "BMI" or "waist circumference" or "metabolic syndrome" or "MS" or "obesity" or "SBP" or "blood pressure" or "DBP" or "fasting glucose" or "FBG" or "TG" or "triglycerides" or "HDL-C" or "HDL cholesterol" for detailed searches.

（3）English Database

We used the keyword AND keyword method for the search. Keyword 1 was set to "taekwondo" and keyword 2 was set to "BMI" or "waist circumference" or "metabolic syndrome " or "MS" or "obesity" or "SBP" or "blood pressure" or "DBP" or "fasting blood glucose" or "FBG" or "TG" or "triglycerides" or "HDL-C" or "HDL cholesterol" were searched.
